# Anesthetic Management of Metastatic Paraganglioma With Spinal Metastases in a Comorbid Cardiovascular Patient

**DOI:** 10.7759/cureus.85044

**Published:** 2025-05-29

**Authors:** Anna Guo, Eric Denisiuk, Kimmy Bais

**Affiliations:** 1 Anesthesiology, University of Toledo College of Medicine and Life Sciences, Toledo, USA; 2 Anesthesiology, The Ohio State University Wexner Medical Center, Columbus, USA

**Keywords:** academic anesthesiology, extra-adrenal paraganglioma, heart failure with reduced ejection fraction, hemodynamic monitoring, neuromonitoring

## Abstract

Paragangliomas are rare neuroendocrine tumors characterized by the secretion of catecholamines. The primary treatment of these tumors consists of a period of medical optimization with oral alpha-blockade followed by surgical resection. Anesthetic management of these patients is challenging due to the potential for significant hemodynamic instability intraoperatively. This report outlines the anesthetic considerations of a patient undergoing multilevel spinal tumor resection of symptomatic metastatic paraganglioma. We present the case of a 55-year-old male patient with a history of heart failure with reduced ejection fraction (HFrEF) who underwent multilevel resection of metastatic paraganglioma of the spine. The patient was treated with one week of oral alpha-blockade preoperatively for uncontrolled hypertension. A combination of low-dose isoflurane, propofol, dexmedetomidine, and remifentanil was used to maintain adequate anesthetic depth while allowing for neuromonitoring. Due to the high level of alpha-blockade, anesthesia-induced hypotension was primarily managed with vasopressin. The intraoperative course was complicated by significant surgical bleeding, which was treated with aggressive volume resuscitation. In the event of hypertension or tachycardia, nicardipine, nitroglycerin, and esmolol were available, although their use was not required. Spinal tumor resection of metastatic paraganglioma requires a thoughtful anesthetic strategy and can be further complicated by patient comorbidities. These conditions require clinicians to thoroughly understand the disease and consider the mechanisms of action of common medications used in anesthesia practice to ensure patient safety.

## Introduction

Paragangliomas are rare neuroendocrine tumors with an overall estimated incidence of one per 300,000, with the incidence of diagnosis on the rise [1,2]. The mean age of diagnosis for patients with sporadic cases is 37, while familial cases are typically diagnosed earlier [3]. They arise from paraganglia cells, a type of neuroendocrine cell that is either extra-adrenal or adrenal in origin. Paragangliomas are tumors of these tissues that are typically extra-adrenal and benign, although about 10% of these cases can spread to organs such as bones, lymph nodes, liver, and lungs [1].

Extra-adrenal tumors are further subdivided into parasympathetic and sympathetic in origin. Tumors located in the neck and skull base are primarily parasympathetic and do not typically secrete catecholamines. Sympathetic paragangliomas, on the other hand, are outside the head and neck, often along the sympathetic chain from the skull base to the bladder and prostate. These paragangliomas are active in norepinephrine secretion, in contrast to closely related pheochromocytoma, which primarily secrete epinephrine. Secretion of hormones leads to symptoms such as hypertension, palpitations, headaches, and sweating [3].

Besides catecholamine secretion, these tumors may cause bony erosion due to their mass effect. In spinal metastases, patients can experience intractable local pain and are at risk for spinal cord compression [4]. Prior to surgery, medical optimization is imperative as patients are at high risk for hypertensive crises due to catecholamine surges, which can occur during manipulation of tumor tissue [4,5]. Blood pressure control is commonly achieved with alpha-blocking agents such as phenoxybenzamine (a nonselective alpha-1 antagonist) or others, such as doxazosin, prazosin, and terazosin (selective alpha-1 antagonists) for a period of 1-3 weeks [5].

## Case presentation

We present a case of a 55-year-old male patient (106.6 kg, BMI: 30.2) with metastatic paraganglioma involving the bladder, prostate, and spine with both intraosseous and extraosseous infiltration of C7, T1, T4, T11, and L1-L4. His medical history was notable for prolonged QT syndrome (QTc 547), asthma, thrombocytopenia (preoperative platelet count of 107 on the morning of surgery), chronic kidney disease (glomerular filtration rate (GFR): 51; creatinine: 1.56 on the morning of surgery), well-controlled gastroesophageal reflux disease, and congestive heart failure with reduced ejection fraction (HFrEF) of 32% (normal range: 50%-70%) due to nonischemic, dilated cardiomyopathy. The patient was admitted for multistage spinal tumor resection due to lower cervical and upper thoracic radiculopathy resulting in symptoms of neck and right-hand pain as well as strength and sensory changes in both lower extremities. Approximately one year prior, the patient underwent uneventful L1 and L3 posterolateral fusion and decompression for an L2 lesion, but the tumor had progressed significantly and resulted in hardware failure. During this admission, he was booked for extensive reconstruction of his lumbar spine including removal of hardware from L1-L4, complete L2 corpectomy, L1-L3 discectomy, and L1-L4 laminectomy as well as L1-L4 arthrodesis with pedicle screw fixation. This was followed by right and left paraspinous muscle flaps, right and left latissimus dorsi myocutaneous flaps, and wound vacuum placement before final closure by plastic surgery.

Upon admission, the patient was found to be hypertensive with systolic blood pressures ranging from the 140s to 170s. He was started on oral doxazosin at an initial dose of 2 mg daily with escalation to 8 mg twice daily over the course of the week leading up to his procedure. Before induction, his mean arterial pressure (MAP) values ranged from approximately 75 to 80 mmHg.

The anesthetic plan for the case was developed in conjunction with the neurosurgical team. Significant hemodynamic swings were anticipated due to mechanical manipulation of tumor tissue with subsequent catecholamine release, as well as from surgical blood loss due to resection of vascularized tumor tissue from the poorly compressible bony structures of the spine. Due to the patient’s alpha-blockade, vasopressin was selected as the first-line pressor to treat hypotension from reduced vascular tone, with epinephrine and norepinephrine available to augment cardiac output in the setting of reduced ejection fraction due to their action as beta-1 receptor agonists. Nicardipine, nitroglycerin, and esmolol were all available in bolus doses as needed in the event of catecholamine-induced hypertensive episodes. The patient had a preoperative blood type and antibody screen completed in anticipation of significant blood loss. Hemodynamic monitoring was achieved invasively with an arterial line placed in the left radial artery prior to induction of anesthesia, allowing for both constant blood pressure monitoring and the ability to draw arterial blood gases (ABGs).

Anesthesia was induced with 180 mg of propofol, 100 mg of 2% lidocaine, 100 mcg of fentanyl, and 100 mg of succinylcholine to achieve optimal intubating conditions. A total of 10 mg of dexamethasone was also administered per the request of the surgeon. The airway was visualized with a McGrath video laryngoscope and secured with an 8.0 mm endotracheal tube. Anesthesia was further maintained with a combination of propofol infusion of 25 mg/kg/minute, dexmedetomidine infusion of 0.2 mcg/kg/hour, remifentanil infusion at 0.05 mcg/kg/minute, and 0.5 MAC of isoflurane to accommodate somatosensory evoked potential (SSEP) and electromyography (EMG) neurological monitoring at the request of the surgical team. Long-acting neuromuscular blockade was contraindicated due to the need for EMG monitoring, necessitating the use of succinylcholine rather than rocuronium for intubation. Infusions were titrated both to hemodynamics (MAP goal of 80 and above) and anesthetic depth as measured by SSEP signal intensity changes, as well as patient movement. Vascular access was established with two 16-gauge peripheral IVs, an 18-gauge peripheral IV, and an 8 French double-lumen central line placed in the right internal jugular vein for infusion of vasoactive medications.

Throughout the procedure, the primary hemodynamic challenge encountered was hypotension caused by a combination of decreased vascular tone from anesthesia and hypovolemia due to blood loss. Significant bleeding began early into tumor resection and persisted throughout the entire case. Bleeding was monitored via suction output, saturated lap pad counts, and serial ABGs. The patient's starting hemoglobin was 13.6. By the end of the 11 hours of surgical time, the estimated blood loss was calculated to be approximately 8,000 mL requiring a transfusion of 27 units of blood products (13 units of red blood cells, 12 units of plasma, and 2 units of platelets totaling approximately 6750 mL) as well as 5500 mL of crystalloid and 1500 mL of albumin. Transfusions were guided by ABG values drawn every 45 minutes to one hour to monitor base excess, lactate, and hemoglobin levels. An intraoperative hemoglobin level of 7 and above was targeted throughout the procedure and successfully maintained. The peak intraoperative lactate level was 1.3 mmol/L (normal range: 0.5 to 1.6 mmol/L), and the peak base deficit was -4.5 mmol/L (normal range: -3.0 to 3.0 mmol/L).

Infusions of vasoactive agents were limited to vasopressin at 0.04 units per minute with additional intermittent boluses of vasopressin of 0.5-1 units for intermittent hypotension. No infusions or boluses of epinephrine or norepinephrine were required during the case. There were no episodes of prolonged hypertension, including during periods of tumor manipulation and resection. Intraoperatively, MAP values ranged from 70 to 85, with a peak systolic blood pressure of 148 briefly occurring once during a stimulating portion of the procedure. Heart rates generally ranged from 60 to 90, with a peak of 117 during the same period of stimulation. Both values quickly normalized with a 30 mg bolus of propofol.

Postoperatively, the patient remained intubated and was transported to the intensive care unit. He was extubated early the next day and proceeded with an uncomplicated recovery. He was subsequently discharged to a rehabilitation facility on postoperative day 8 with subjective improvement of strength and sensation in his left lower extremity.

## Discussion

Paraganglioma resection poses a unique challenge to anesthesiologists, especially in cases of spinal metastases. Patients are at high risk of hemodynamic swings from catecholamine-induced hypertension, postresection hypotension from catecholamine depletion, and anesthesia-induced hypotension [6]. In addition, paragangliomas are highly vascularized tumors that can lead to significant intraoperative blood loss, causing hemodynamic instability and often necessitating blood transfusion [3]. Patient comorbidities can further complicate management and must be considered when formulating the anesthetic plan.

In the setting of preoperative alpha-blockade, many vasopressors such as phenylephrine, epinephrine, and norepinephrine have reduced effectiveness in treating hypotension as they all act on alpha-receptors. As a result, the primary methods of maintaining hemodynamic stability included aggressive volume resuscitation to offset surgical intravascular volume loss, using the lowest possible doses of anesthetic medications to minimize vasodilatory hypotension, and selection of functional pressors and inotropes to augment vascular tone and cardiac output. In this case, fluid losses totaled approximately 8,700 mL (8,000 mL of blood and 700 mL of urine), and resuscitation was achieved with approximately 13,750 mL of combined blood products, crystalloid, and albumin for a net positive volume of 5,050 mL. Vasopressin was chosen as a first-line agent due to its action on V1 receptors and the absence of alpha-receptor activity, and its continuous infusion with intermittent bolusing proved to be adequate for maintaining the respective goal MAP and organ perfusion, as evidenced by normal intraoperative lactate levels. Epinephrine and norepinephrine were available as additional inotropic agents to improve cardiac output in the event of refractory hypotension, but neither was required during this case [7,8].

Other hemodynamic concerns included the possibility of hypertensive emergency and tachycardia during resection/manipulation of tumor tissue. In the setting of anticipated reduced preload from surgical volume losses, short and rapid-acting vasodilators were selected. Nicardipine was chosen as the first-line agent due to its selectivity for arterial vasodilation in consideration of the patient’s history of HFrEF. Nitroglycerin, primarily a venodilator, was available as a second-line agent. Esmolol, a short-acting beta-blocker, was the preferred agent for acute tachycardia [7,8]. Fortunately, due to the patient’s excellent presurgical optimization, no such hypertensive or tachycardic events occurred.

## Conclusions

This report documents the nuances and careful considerations of anesthetic management in a patient undergoing spinal tumor resection of metastatic paraganglioma. This case was difficult due to the extensive involvement of the paragangliomas, prior surgeries, cardiovascular comorbidities of the patient, perioperative alpha-blockade, and continuous neurological monitoring. A judicious approach involving a careful balance of volume transfusion, IV and inhalational anesthetic agents, and careful cardiovascular monitoring is important to ensure patient safety and the best possible surgical outcomes.
